# Biocompatible Mesoporous Hollow Carbon Nanocapsules for High Performance Supercapacitors

**DOI:** 10.1038/s41598-020-61138-4

**Published:** 2020-03-09

**Authors:** Lijian Wang, Fenghua Liu, Yuesheng Ning, Robert Bradley, Chengbin Yang, Ken-Tye Yong, Binyuan Zhao, Weiping Wu

**Affiliations:** 1 State Key Laboratory of Metal Matrix Composites, School of Materials Science and Engineering, Shanghai Jiao Tong University, Shanghai, 200240 China; 2 Department of Materials, University of Oxford, 16 Parks Road, Oxford, OX1 3PH United Kingdom; 3 MatSurf Ltd, The Old Stables Marion Lodge, Little Salkeld, Penrith, Cumbria CA10 1NW United Kingdom; 4 School of Energy Resources, University of Wyoming, Laramie, WY 82071 USA; 5 Guangdong Key Laboratory for Biomedical Measurements and Ultrasound Imaging, School of Biomedical Engineering, Health Sciences Center, Shenzhen University, Shenzhen, 518060 China; 6 School of Electrical and Electronic Engineering, Nanyang Technological University, Singapore, 639798 Singapore; 7 Department of Electrical and Electronic Engineering, School of Mathematics, Computer Science and Engineering, City, University of London, Northampton Square, London, EC1V 0HB United Kingdom

**Keywords:** Electrical and electronic engineering, Biomaterials - cells, Porous materials

## Abstract

A facile and general method for the controllable synthesis of N-doped hollow mesoporous carbon nanocapsules (NHCNCs) with four different geometries has been developed. The spheres (NHCNC-1), low-concaves (NHCNC-2), semi-concaves (NHCNC-3) and wrinkles (NHCNC-4) shaped samples were prepared and systematically investigated to understand the structural effects of hollow particles on their supercapacitor performances. Compared with the other three different shaped samples (NHCNC-1, NHCNC-2, and NHCNC-4), the as-synthesized semi-concave structured NHCNC-3 demonstrated excellent performance with high gravimetric capacitance of 326 F g^−1^ (419 F cm^−3^) and ultra-stable cycling stability (96.6% after 5000 cycles). The outstanding performances achieved are attributed to the unique semi-concave structure, high specific surface area (1400 m^2^ g^−1^), hierarchical porosity, high packing density (1.41 g cm^−3^) and high nitrogen (N) content (up to 3.73%) of the new materials. These carbon nanocapsules with tailorable structures and properties enable them as outstanding carriers and platforms for various emerging applications, such as nanoscale chemical reactors, catalysis, batteries, solar energy harvest, gas storage and so on. In addition, these novel carbons have negligible cytotoxicity and high biocompatibility for human cells, promising a wide range of bio applications, such as biomaterials, drug delivery, biomedicine, biotherapy and bioelectronic devices.

## Introduction

Supercapacitors have drawn increasing attention due to their high power density, fast charge/discharge capability, long cycle life, low cost and environmental friendliness among various electrochemical energy storage devices^1^. Carbon materials are the most promising supercapacitor electrode materials, due to their high specific surface area, high electrical conductivity, tailorable pore structures and excellent chemical stability^2^. The pore structures and the surface properties of carbon materials have significant effects on the electrode performances^3^. In order to increase the volumetric capacitance of the electrode materials^4,5^, it is vital to optimize the morphology and reduce the void volume of the hollow porous carbon materials.

Hollow carbon nanocapsules (HCNCs) are an interesting class of carbon materials, due to their unique properties such as high surface-to-volume ratio, low densities, large void space and high electrical conductivity^6,7^. The hollow macropore cavities and interior space of the HCNCs can act as anion-buffering reservoirs to shorten the diffusion distance of electrolyte ions^8^, while the mesopores and micropores in the shell can promote ion transport, minimize diffusion pathways from the electrolyte to the micropores, and increase the specific surface area to maximize the electric double layer capacitance^9^. For high-rate performances, HCNCs are particularly interesting as they have ultrahigh specific surface area, large surface-to-volume ratio, large void space along with an appropriate interconnected pore channels to facilitate ion diffusion^10^. Previously, hollow carbon nanocapsules with unique structures such as yolk-shell spheres^11^, multi-shelled hollow spheres^12^, vesicles^13^, nanotubes^14^ and many other shaped carbon materials have been prepared. However, hollow carbons with unique geometry, architectures or shapes have not been reported, due to the lack of facile and general sythesis methods available. Besides, these materials with 3D sphere hollow structures reported, usually have defects or voids that occupy much space, while the sizes and thicknesses of the particles are nonuniform as these parameters are difficult to control^15,16^.

On the other hand, undoped carbon materials have hydrophobic surfaces and a limited energy density, due to their limited electrochemical double layer capacitive (EDLC) storage capability, which impedes their practical applications^17^. In this respect, the introduction of heteroatoms (such as B, N and S) onto the surface or into the bulk of the carbon materials, is a promising approach to improve the specific capacitance and energy density by the extra surface faradaic reactions^18–20^. In particular, N-doped carbon materials with hierarchical porous structures are promising because of their lower atomic radii and higher electronegativity^21^. Furthermore, pyridine-N and pyrrolic-N could provide active sites for electrode materials to enhance the electrical activity, promoting electron transfer, leading to excellent pseudocapacitance of carbon materials^22,23^.

In this paper, we present a facile, controllable one-step method of synthesizing N-doped hollow porous carbon nanocapsules (NHCNCs) with four different morphologies in a single synthetic system. Simply by changing the ratio between carbon precursors and tetraethyl orthosilicate (TEOS), the structures of the obtained hollow carbon materials can be controllably tuned from hollow spheres, low-concaves, semi-concaves to wrinkled disks. The cavity volume of the hollow carbon nanocapsules also gradually decreases. Particularly, compared with the other three shapes (NHCNC-1, NHCNC-2, and NHCNC-4), the as-synthesized NHCNC-3 with semi-concave structure has an excellent balance between pore volume and compact density, resulting in excellent electrochemical performances. This new nanoscale material architecture combines several advantages and benefits for electrode applications: (1) better connection with neighboring particles and thinner shell with mesoporous structures, resulting into reduced ion-transport resistance which enables ion transport at high electrical current density; (2) hierarchical porous structures; (3) lower void volume showing increased mass density due to their better stacking behaviour which will greatly contribute to volumetric capacitance; (4) relatively high content of nitrogen heteroatoms could provide a certain amount of pseudocapacitance. These characteristics enable the NHCNC-3 to have outstanding gravimetric capacitance and volumetric capacitance for supercapacitors. This work provides a good example on the correlation between carbon nanocapsule structures and their electrochemical performances.

## Results

**Figure 1:**
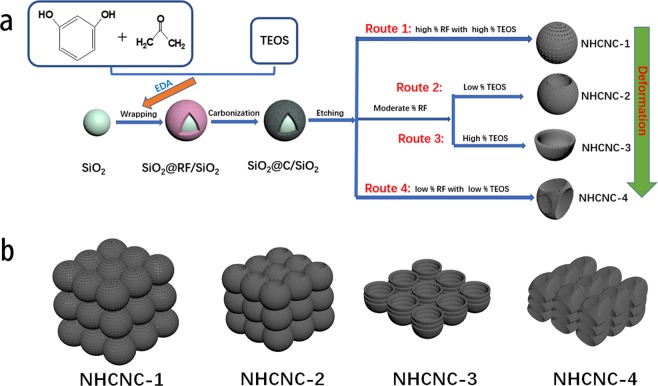
(**a**) Schematic synthesis processes of the hollow porous carbon nanocapsules (NHCNCs) with different morphologies, (**b**) the stacking behaviours of the four NHCNCs.

The synthesis strategy of N-doped hollow porous carbon nanocapsules with different morphologies was depicted in Fig. 1a. Silica (SiO_2_) spheres with a uniform diameter of ~180 nm were wrapped with a layer of the resorcinol-formaldehyde polymer by a one-pot polymerization. Ethylenediamine (EDA), a catalyst for both the polymerization and tetraethyl orthosilicate (TEOS) hydrolysis, was also used as the nitrogen (N) precursor for *in-situ* N doping in the carbon materials.

After the carbonization, and etching of the SiO_2_ spheres as well as small SiO_2_ particles intertwined within the carbon layers, NHCNCs were successfully prepared. In particular, hollow carbon nanocapsules with different morphologies can be controlled by adjusting the content of phenolic resin and TEOS. Specifically, the thickness of the carbon shell could be changed by adjusting the phenolic resin concentrations. TEOS, as a pore-forming agent, also plays a crucial role in the morphological changes of NHCNCs. Apparently, at a high phenolic resin concentration, the regular spherical NHCNCs with a thick carbon shell will be formed, regardless of the content of TEOS being added (Route 1). As the phenolic resin concentration decreases, the thinner porous carbon shells could not maintain the original spherical structure and they collapse inward to form low-concave (Route 2, with low% TEOS) or semi-concave (Route 3, with high% TEOS) structure. For NHCNCs with ultra-thin carbon shell, even if a small amount of TEOS was added, the original spherical structure collapses into a wrinkled structure immediately after silica was removed (Route 4). Besides, different structures of NHCNCs exhibit different stacking behaviours (Fig. 1b). Compared to the hollow spherical structures, the carbon shell collapsed into its internal cavity could lead to more compact stacking between particles and increase the mass density. The NHCNC-3 with a unique semi-concave structure and relatively small cavity volume is more likely to be tightly stacked. This would be very important and useful to increase their packing density and achieve higher volumetric capacitance.

**Figure 2:**
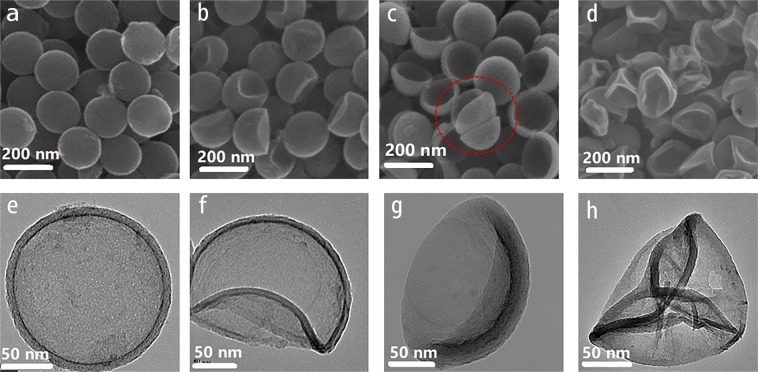
Scanning Electron Microscopy (SEM) images of (**a**) NHCNC-1, (**b**) NHCNC-2, (**c**) NHCNC-3 and (**d**) NHCNC-4; Transmission Electron Microscopy (TEM) images of (**e**) NHCNC-1, (**f**) NHCNC-2, (**g**) NHCNC-3 and (**h**) NHCNC-4.

The morphology of as-synthesized NHCNCs was observed by Scanning Electron Microscope (SEM) and Transmission Electron Microscope (TEM). As shown in Fig. 2a,d, the highly uniform NHCNCs with average diameters of 180 ± 10 nm can be observed. After the removal of the silica component, the spherical structure of NHCNC-1 with a shell thickness of 15 nm can be well maintained (Fig. 2a,e). For the other three different types of NHCNCs (NHCNC-2, NHCNC-3, NHCNC-4), the original spherical shape gradually deformed with the change of the carbon precursor and TEOS concentrations. As the concentration of carbon precursor decreases, the remaining carbonaceous framework of NHCNCs after removal of SiO_2_ will not be mechanically robust or stable enough, so that the carbon shell collapses into the hollow interior to form unique low-concave shapes (Fig. 2b,f). As the amount of TEOS increased from 0.15 mL to 0.45 mL, the low-concave shape of NHCNC-2 further collapsed into the semi-concave shape of NHCNC-3 (Fig. 2c,g).

**Figure 3:**
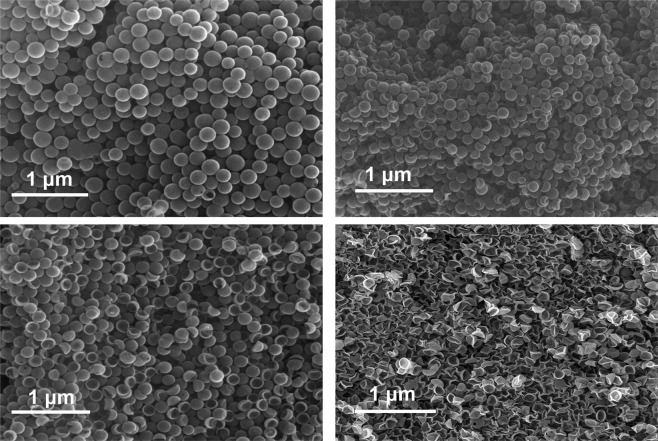
Low magnification Scanning Electron Microscopy (SEM) images of (**a**) NHCNC-1, (**b**) NHCNC-2, (**c**) NHCNC-3, (**d**) NHCNC-4.

By using lower concentration of carbon precursors, the shell thickness of NHCNC-2 and NHCNC-3 became smaller than NHCNC-1, which may be beneficial for ion transmission^23^. Compared to NHCNC-2, the more amount of TEOS pore-forming agent may cause NHCNC-3 to have more porosity and larger pore size, the void volume inside NHCNC-3 is drastically reduced, which may increase the bulk density of the material, leading to higher specific capacitance^24^. However, when the concentration of the carbon precursor is further reduced, even with less TEOS, the hollow carbon nanocapsules will collapse into a wrinkled shape after the silica core and shell framework were removed (Fig. 2d,h). In a word, we can fully control the synthesis of hollow porous carbon nanocapsules with different morphologies by adjusting the thickness of the carbon shell and the amount of TEOS. The obtained hollow porous carbon nanocapsules show a good level of uniformity, high density packing, forming continuous conductive networks on the surfaces of electrodes (Fig. 3a–d).

**Figure 4:**
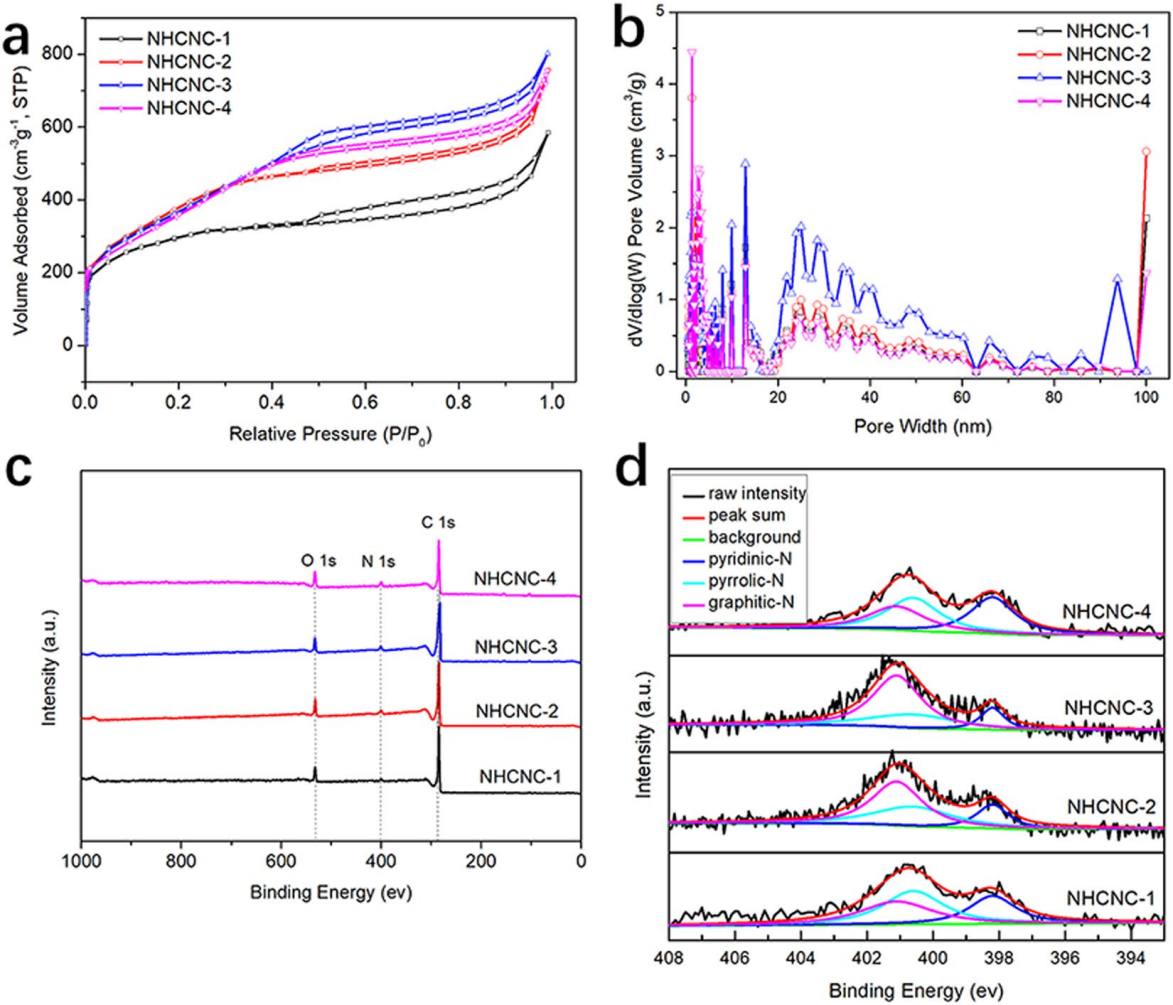
(**a**) Nitrogen adsorption-desorption isotherms, (**b**) NLDFT model pore size distributions, (**c**) X-ray Photoelectron Spectroscopy (XPS) survey spectrum, (**d**) N1s XSP spectrum of NHCNCs.

The nitrogen (N_2_) isothermal adsorption-desorption isotherms were performed to analyse the textural properties of the carbon nanocapsules. An IV type isotherm and an H4-type hysteresis loop were observed from the nitrogen (N_2_) adsorption-desorption curves of NHCNCs (Fig. 4a). Specifically, three typical regions can be observed: (i) a sharp increase at low pressure (P/P_0_ < 0.01) confirms the presence of micropores; (ii) when P/P_0_ is increased from 0.4 to 0.9, a large hysteresis loop in the isotherm curves indicates the existence of mesopores; and (iii) when relative pressure went up close to 1.0, a steep rise is also observed, which may be attributed to a large cavity in hollow nanocapsules formed by the removal of silica. In particular, the hysteresis loops of NHCNC-3 tend to be wider than NHCNC-2 and NHCNC-4, suggesting more mesopores in the carbon nanocapsules.

The pore size distribution was calculated using the NLDFT model (Fig. 4b). The pore size distribution curves of the four samples have a large number of peaks from 0.56 to 100 nm, indicating the existence of micropores/mesopores/macropores in the samples. The gradual broadening of meso/macropores from 20 nm to 100 nm is consistent with the trend of increasing TEOS and reducing carbon shell thickness, confirming the pore diameters of NHCNCs can be well controlled. TEOS, as a pore-forming agent, uniformly dispersed in the phenolic resin, which resulted in high porosity and high specific surface area. When reducing the proportion of phenol resin, the high amount of TEOS would result in more and larger silica participated in the carbon precursors. As a result, the specific surface area and pore diameter of carbon materials increased. NHCNC-3 has the largest surface area (1400.96 m^2^ g^−1^) as well as pore volume (1.6079 cm^3^ g^−1^), suitable pore diameter (2.25 nm), which indicates that the materials have more spaces and surface-active sites for electrochemical processes in the supercapacitors.

To further determine the chemical composition of NHCNCs, X-ray Photoelectron Spectroscopy (XPS) analysis was conducted (Fig. 4c,d). The XPS spectrum confirms the existence of C, O, and N elements in the carbon materials. As shown in Supplementary Table S1, the nitrogen (N) contents for NHCNC-1, NHCNC-2, NHCNC-3 and NHCNC-4 are 2.84 at%, 3.45 at%, 3.73 at%, and 4.51 at%, respectively. The high N contents of NHCNCs are attributed to the EDA introduced into the reaction system, which was used as a catalyst and the precursor supplying the nitrogen element for the *in-situ* N doping in the NHCNCs. The XPS spectrum of N1s can be fitted into three different nitrogen groups at 398.2 eV, 400.6 eV, 401.1 eV (Fig. 4d), corresponding to pyridinic N, pyrrolic N, graphitic N^25^. Pyridine-N and pyrrolic-N are electrochemically active in electrode materials with enhanced electrochemistry activity and pseudocapacitance^23^. The graphitic-N can effectively promote electron transfer of carbon materials. Meanwhile, the XRD and Raman spectroscopy confirm that these carbon materials are composed of both crystalline and amorphous components (Supplementary Figs. S1, S2). The high surface area, adjustable internal structure, a large cavity, and suitable N-doping make the NHCNCs be the ideal electrode materials for supercapacitors.

**Figure 5:**
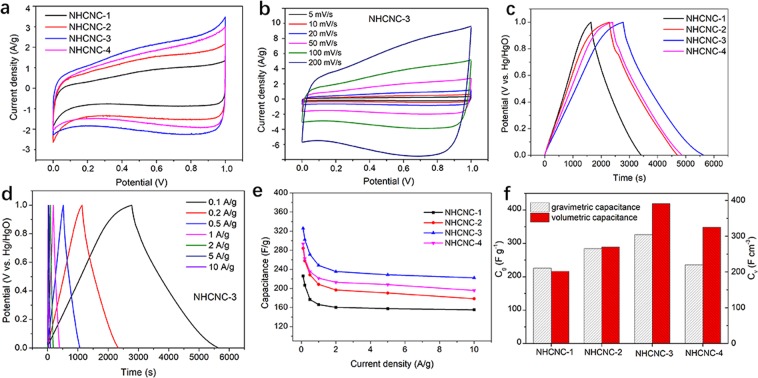
Electrochemical performances of NHCNCs in 6 M KOH electrolyte: (**a**) Cyclic Voltammetry (CV) curves at 5 mV s^−1^ for all NHCNCs; (**b**) CV curves at various scan rates for NHCNC-3; (**c**) Galvanostatic charge-discharge (GCD) curves at 0.1 A g^−1^ for all NHCNCs; (**d**) GCD curves at versus charge/discharge current density for NHCNC-3; (**e**) Corresponding gravimetric capacitance versus current density from 0.1 A g^−1^ to 10 A g^−1^; (**f**) Volumetric capacitances vs. gravimetric capacitances of NHCNCs at 0.1 A g^−1^.

In order to investigate the effect of the morphology of hollow carbon nanocapsules on electrochemical energy storage, the NHCNCs were tested as supercapacitor electrode materials using a three-electrode electrochemistry system. The Cyclic Voltammetry (CV) curves of all NHCNCs at 5 mV s^−1^ (Fig. 5a) are quasi-rectangular, which can be explained by two different capacitances comprising the dominate electric double layer capacitance (EDLC) and the less faradaic pseudocapacitance derived from doping of heteroatoms^11^.

The larger raised regions in the CV curves are contributed by the pseudocapacitance resulting from the doping of nitrogen and oxygen atoms. Obviously, the largest area in the CV curves of NHCNC-3 compare to all samples indicates its superior specific capacitance for energy storage (Fig. 5a). The CV curves of all NHCNCs were also tested at scan rates from 5 to 200 mV s^−1^ (Fig. 5b and Supplementary Fig. S3a–c). With the increase of scan rate, the rectangular shape appears slightly deformed due to the influence of the heteroatoms contained in the NHCNCs.

All the galvanostatic charge-discharge (GCD) curves show similar isosceles triangular shapes (Fig. 5c**)**, even at high current densities 10 A g^−1^ **(**Fig. 5d and Supplementary Fig. S3d–f**)**, indicating typical supercapacitor behaviour and superior reversibility of charge-discharge processes. The spherical carbon nanocapsules (NHCNC-1) have the lowest gravimetric capacitance (*C*_g_), only 226.1 F g^−1^ at 0.1 A g^−1^. As both the void volume and the shell thickness decrease, the *C*_*g*_ of the other three samples increases.

The capacitance of NHCNC-2, NHCNC-3, NHCNC-4 were 284.2 F g^−1^, 326 F g^−1^, 293.4 F g^−1^, respectively (Fig. 5c**)**. In agreement with the CV results, the NHCNC-3 exhibits the largest gravimetric capacitance among these four hollow carbon materials, which is the highest among recently published carbon materials, such as graphene^26^, CNFs^27^, MWNT^28^ and carbon aerogels^29^ (Supplementary Table S2). At high current density (10 A g^−1^), the capacitor retention of NHCNC-3 is 76.5%, showing good rate performance (Fig. 5e). The high gravimetric capacitance of NHCNC-3 may be due to its high specific surface area, ideal pore size distribution, small hollow shell thickness, and moderate nitrogen atom doping. Moreover, compared to the other three hollow structures, its unique semi-concave structure enables closer connections between adjacent particles to increase the contact area, resulting in enhanced charge transfer and improved capacitance performance.

The volumetric capacitance (*C*_*v*_) is also an important parameter for evaluating electrochemical performance, especially in compact energy storage devices^30^. The packing densities (*ρ*_*v*_) of NHCNCs were determined by the reported method **(**Supplementary Fig. S4**)**. The spherical structured NHCNC-1 with a larger hollow volume has the least packing density (0.96 g cm^−3^), resulting in a smaller volumetric capacitance of 216.4 F cm^−3^ (according to *C*_*v*_ = *C*_*g*_ × *ρ*_*v*_) shown in Fig. 5f. Notably, as the hollow spherical structure collapses into the semi-concave structure (NHCNC-3), the original hollow volume shrunk, leading to a better stack. Both higher packing density (1.41 g cm^−3^) and higher volumetric capacitance (419 F cm^−3^) were achieved in the sample NHCHC-3. Further decreasing the carbon thickness, however, led to a wrinkled structured NHCNC-4 and irregular stacking, which will degrade the capacitance. Interestingly, the volumetric capacitance of NHCNC-3 is found to be about 5 times of CNCs^6^ and one of the highest among all carbon materials developed up to date^14^ (Supplementary Table S2). Therefore, this strategy makes it possible for hollow carbon materials to achieve both higher gravimetric capacitance and higher volumetric capacitance.

## Discussion

**Figure 6:**
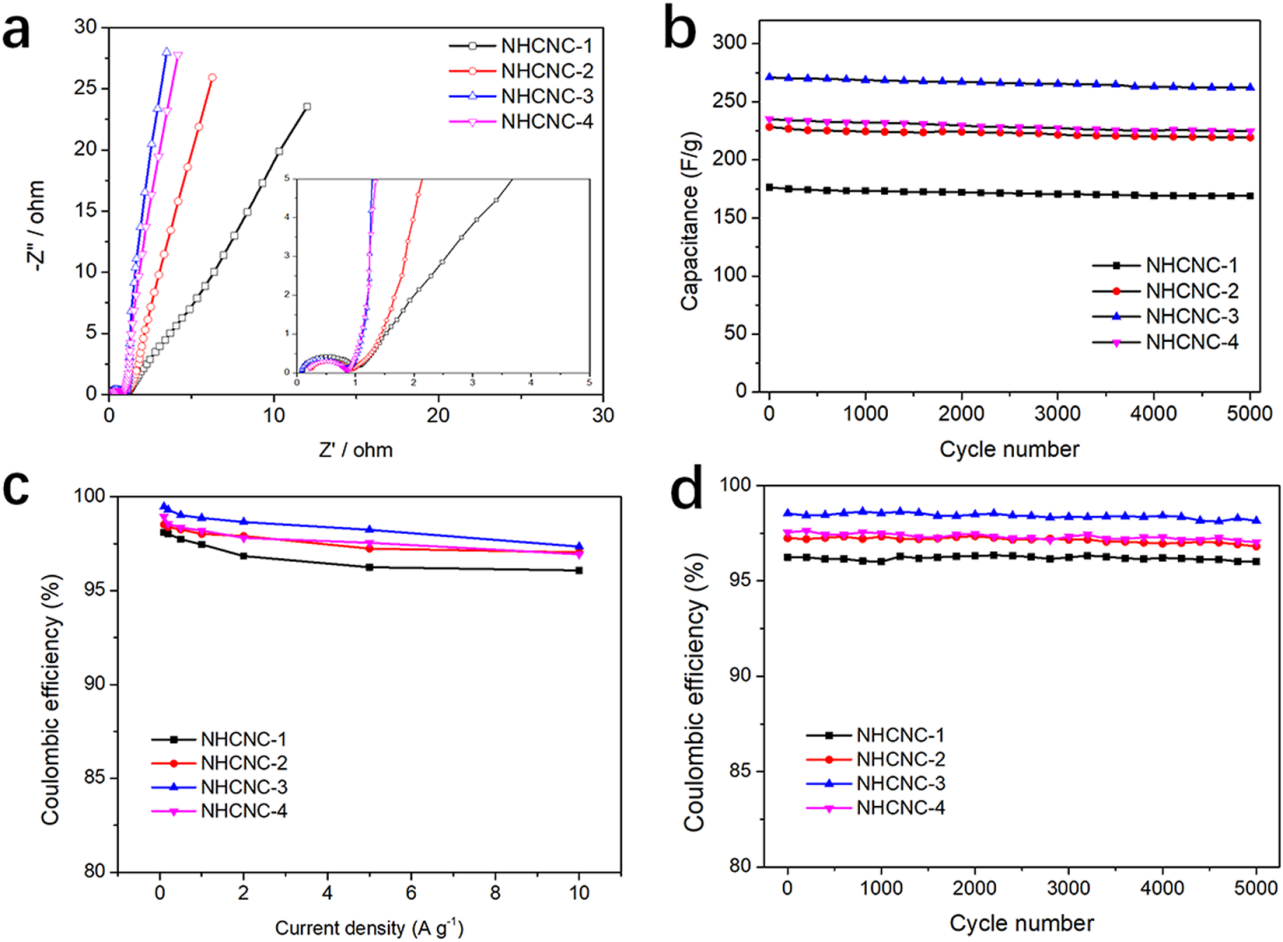
(**a**) The Nyquist plots of NHCNCs (0.1 Hz to 100 kHz), inserted is the enlarged spectrum in the low impedance regime, (**b**) Cycling performance of NHCNCs (5.0 A g^−1^ for 5000 cycles). (**c**) Coulombic efficiency of NHCNCs at different current densities, (**d**) Coulombic efficiency for 5000 cycles at a current density of 5.0 A g^−1^.

The electrochemical impedance spectroscopy (EIS) of NHCNCs was measured from 10^−1^ Hz to 10^5^ Hz (Fig. 6a). The equivalent series resistance (ESR) of NHCNC-3 was much lower than the rest three samples. This also indicates that the semi-concave structure of NHCNC particles with thinner shell thickness can tightly connect with adjacent particles compared with spherical and other two structures, which facilitates reducing the interfacial electrical contact resistance and shortens the ion transmission path. The long cycling life is also very important for the practical applications of supercapacitors. As shown in Fig. 6b, all the four NHCHCs have excellent capacitance retention after 5000 cycles (at a current density of 5.0 A g^−1^), indicating their long-term electrochemical stability. The Coulombic efficiencies for all the four materials (NHCNC-1 to NHCNC-4) at different current densities and cycling are more than 95%, which demonstrates the good electrochemical stability of the NHCNCs electrode (Figs. 6c,d). Particularly, the Coulombic efficiency of NHCNC-3 is higher than other three NHCNCs, due to its high surface area and pore structures that makes it easier to fully contact the electrolyte^31^.

Therefore, according to our research, adjusting the structure of hollow carbon nanocapsules can effectively improve the properties of materials, especially as electrode materials for energy storage applications. Comparing the four structures of hollow carbon nanocapsules, the NHCNC-3 with semi-concave structure is more suitable as an electrode material for supercapacitor, as illustrated in Fig. 1. Firstly, the high specific surface area and the hierarchical porous structure are contributing to the high capacitance, high rate performance and cycle stability. Secondly, the suitable nitrogen atoms doped in the carbon materials can improve the wettability, electrochemical activity and provide extra pseudocapacitance. Thirdly, compared to spherical and low-concave counterparts, the semi-concave hollow NHCNC-3 with thinner shell thickness has a small internal cavity, which shortens the diffusion paths of electrolytes to the carbon shells. In addition, the semi-concave particles are more tightly connected to each other than the other three structures, so that the packing density and the volumetric capacitance of the material are increased.

Since NHCHCs possess outstanding performance as supercapacitor materials, it is possible to explore their application in wearable or implantable devices. Before that, it is crucial to assess the biocompatibility and cytotoxicity of the NHCHCs. Hence, the cytotoxicity of NHCNC nanoparticles was evaluated by MTT assay using Hela cell (human cervical cancer cell line)^32^. As shown in Supplementary Fig. S5, both NHCNC-1 and NHCNC-3 show no significant cytotoxicity on HeLa cells after 24 h of co-incubation. The NHCNCs-treated cells remained intact structure, density and shapes on the cell culture dish, and display similar growing status controlling groups (un-treated cell groups). In addition, zeta potentials of NHCNC-1, NHCNC-2, NHCNC-3 and NHCNC-4 were 24.21 mV, 14.87 mV, 10.4 mV and 20.35 mV, respectively, clearly confirming that NHCNCs are not cytotoxic **(**Supplementary Fig. S6**)**^33^. These results clearly indicate that NHCHCs display negligible cytotoxicity and high biocompatibility to human cell lines, which signify their potential application as supercapacitor in wearable and implantable devices.

In conclusion, N-doped hollow mesoporous carbon nanocapsules (NHCNCs) with four different structures in a single synthetic system have been prepared by a facile method. By controlling the synergistic effect between shell thickness and pore structure in the carbon layer, the original spherical structure SiO_2_@C/SiO_2_ can be controllably transformed into hollow carbon nanocapsules as spheres (NHCNC-1), low-concaves (NHCNC-2), semi-concaves (NHCNC-3) and wrinkled disks (NHCNC-4). NHCNCs with different morphologies provide high surface areas, hierarchical mesoporous structures and nitrogen atoms doping, which are beneficial to the high capacitance, good capacitance retention and cycling stability. The semi-concave structured NHCNC-3 with moderate cavity volume has superior gravimetric and volumetric capacitance compared to all the rest of the three carbon materials. This strategy expands the family of hollow carbon nanocapsules with tunable structures, surface areas and chemical elemental doping, making them ideal materials for energy storage applications. These carbon nanocapsules with a tailorable internal cavity volume enable them as outstanding carriers and platforms for various emerging applications, such as batteries, solar energy harvest, nanoscale chemical reactors, catalysis, gas storage and so on. In addition, the cytotoxicity of carbon nanoparticles was evaluated. The negligible cytotoxicity for human cells verified their excellent biocompatibility, promising a wide range of bio applications, such as biomaterials, drug delivery, biomedicine, biotherapy and bioelectronic devices.

## Methods

### Synthesis of N-doped hollow porous carbon nanocapsules

NHCNCs were prepared through a facile route by using SiO_2_ spheres as hard template. The SiO_2_ spheres were synthesized following a slightly modified Stöber process^34^. In a typical procedure to synthesize the NHCNCs^35^, 0.5 grams of SiO_2_ spheres were uniformly dispersed in a premixed solution composed of 30 mL ethanol and 70 mL deionized water. Then, resorcinol, EDA and formaldehyde were added to the above solution at intervals of 5 min under stirring at 35 °C. After 10 min, a certain amount of TEOS (dispersed in 20 ml of ethanol) was added dropwise and the system was maintained stirring at 35 °C for 24 h. The precipitates were separated by centrifugation and dried at 60 °C overnight. The product was then carbonized under N_2_ at 900 °C for 3 h, and the different structures of NHCNCs were obtained after the removal of silica by 10% hydrofluoric (HF) acid solution. In order to synthesize NHCNC-1, NHCNC-2, NHCNC-3 and NHCNC-4, the amount of resorcinol/formaldehyde/TEOS were set at 0.2 g/0.3 mL/0.45 mL, 0.15 g/0.2 mL/0.15 mL, 0.15 g/0.2 mL/0.45 mL and 0.1 g/0.15 mL/0.15 mL.

### Characterizations

The hollow carbon nanocapsules were characterized by scanning electron microscopy (SEM, FEI Sirion200) and transmission electron microscopy (TEM, EM-2100F JEOL). The composition and phase of samples were characterized via XRD (D8 ADVANCE Da Vinci with Cu Kα radiation) operated at 40 kV and 30 mA, X-ray photoelectron spectroscopy XPS (Kratos AXIS Ultra DLD) with a monochromatic AlKα X-ray source and Raman spectra with a Horiba JobinYvon HR 800 Raman spectrometer. The Nitrogen (N_2_) adsorption-desorption isotherms were collected by a TriStar 3000 analyser at 77 K, the pore size distributions were estimated using the Nonlocal Density Function Theory (NLDFT) model. The zeta potentials of NHCNCs were analysed by a NanoBrook Omni Particle Size and Zeta Potential Analyzer (Brookhaven Instrument Corporation, New York, USA).

### Electrochemical measurements

Electrochemical features were tested on CHI 660D electrochemical working station in 6 M KOH electrolyte in a three-electrode configuration. An Hg/HgO electrode and a piece of Pt were served as reference and counter electrodes, respectively. The working electrode was prepared by pressing homogeneous slurry of carbon sample, acetylene black, and polytetrafluoroethylene (weight ratio of 8:1:1) on nickel foam current collector (1 × 1 cm), then dried at 100 °C for 12 hours. The mass loading of active materials was about 2.0 mg cm^−2^. The voltage for CV and GCD varied from 0 V to 1 V and the GCD current densities were from 0.1 to 10.0 A g^−1^. The EIS spectra were obtained in a frequency range from 100 Hz to 0.1 Hz.

## Supplementary information

Supporting information.
