# Supplementary material for: Biocompatible Mesoporous Hollow Carbon Nanocapsules for High Performance Supercapacitors
Source: Sci Rep. 2020 Mar 9;10:4306. doi: 10.1038/s41598-020-61138-4 (PMC7062802; doi:10.1038/s41598-020-61138-4)
Supplement: Supplementary file 1 — Supporting information. [file 41598_2020_61138_MOESM1_ESM.doc]

**Supporting information**

**Biocompatible Mesoporous Hollow Carbon Nanocapsules for High Performance Supercapacitors**

# Lijian Wang^1^, Fenghua Liu^1^, Yuesheng Ning^1^, Robert Bradley^2,3,4^, Chengbin Yang^5,*^, Ken-Tye Yong^6^, Binyuan Zhao^1,*^ and Weiping Wu^7,*^

^1^State Key Laboratory of Metal Matrix Composites, School of Materials Science and Engineering, Shanghai Jiao Tong University, Shanghai, 200240, China

^2^Department of Materials, University of Oxford, 16 Parks Road, Oxford, OX1 3PH, United Kingdom

^3^MatSurf Ltd, The Old Stables Marion Lodge, Little Salkeld, Penrith, Cumbria, CA10 1NW, United Kingdom

^4^School of Energy Resources, University of Wyoming, Laramie, WY 82071, USA

^5^Guangdong Key Laboratory for Biomedical Measurements and Ultrasound Imaging, School of Biomedical Engineering, Health Sciences Center, Shenzhen University, Shenzhen 518060, China

^6^School of Electrical and Electronic Engineering, Nanyang Technological University, Singapore 639798, Singapore

^7^Department of Electrical and Electronic Engineering, School of Mathematics, Computer Science and Engineering, City, University of London, Northampton Square, London, EC1V 0HB, United Kingdom

*Corresponding authors:

E-mail: byzhao@sjtu.edu.cn, cbyang@szu.edu.cn, Weiping.Wu@city.ac.uk

**Contents**

1. XRD characterization results

2. Raman characterization results

3. Electrochemistry performances of the supercapacitor devices with NHCNCs

4. A photograph of 100 mg of NHCNC samples tapped in quartz tubes

5. The cytotoxicity of hollow carbon nanospheres on Hela cell line

6. Zeta potential of the prepared NHCNCs

7. Textural parameters, density and elemental composition

8. Comparison of the NHCNCs with previously reported carbon materials

**1. XRD characterization results**


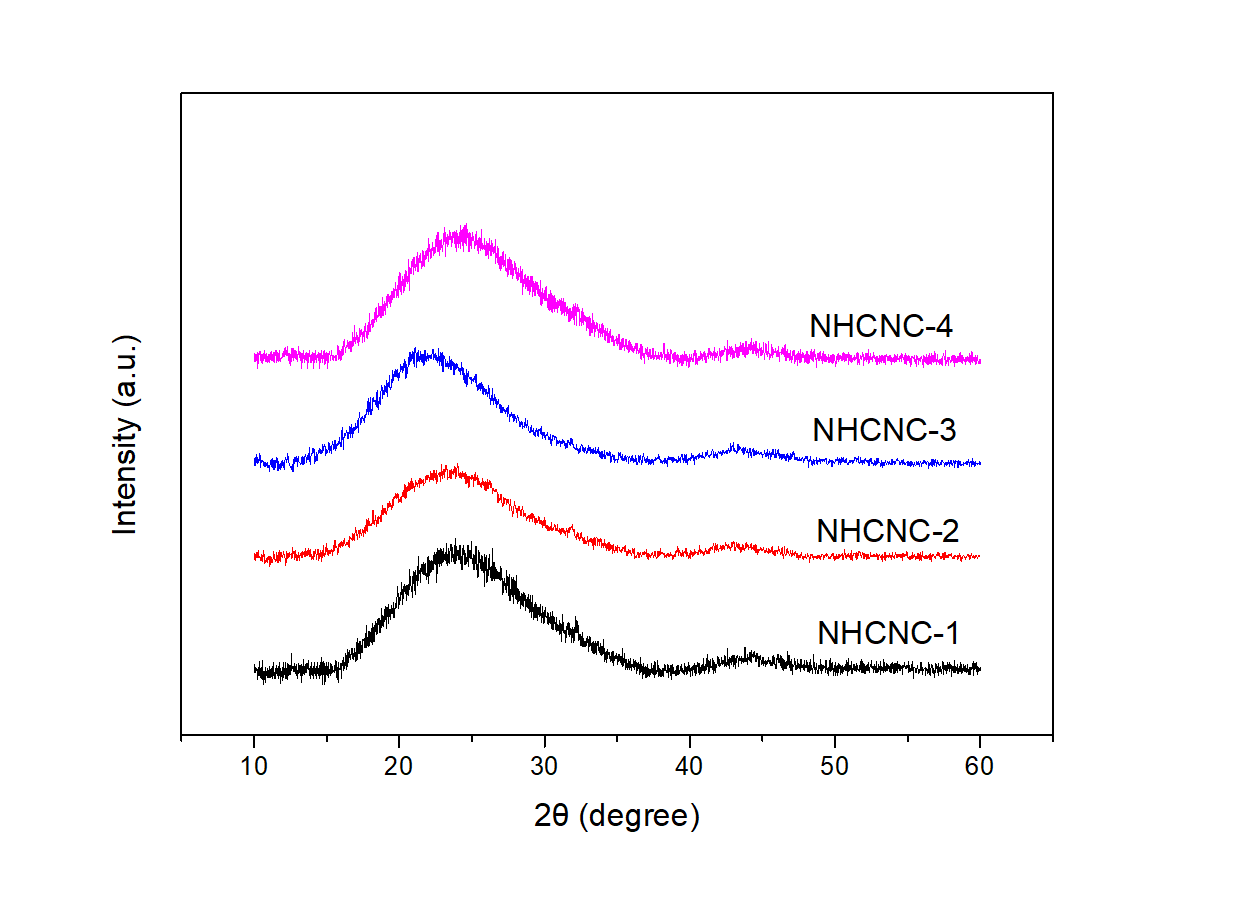


**Figure S1.** The XRD patterns of NHCNCs, a broad intensified peak at 24.7 ^o^ is observed along with a low intensity hump at 43.8 ^o^ for both samples, corresponding to the (002) and (001) *hkl* planes indicating amorphous structure of these carbon samples.

**2. Raman characterization results**


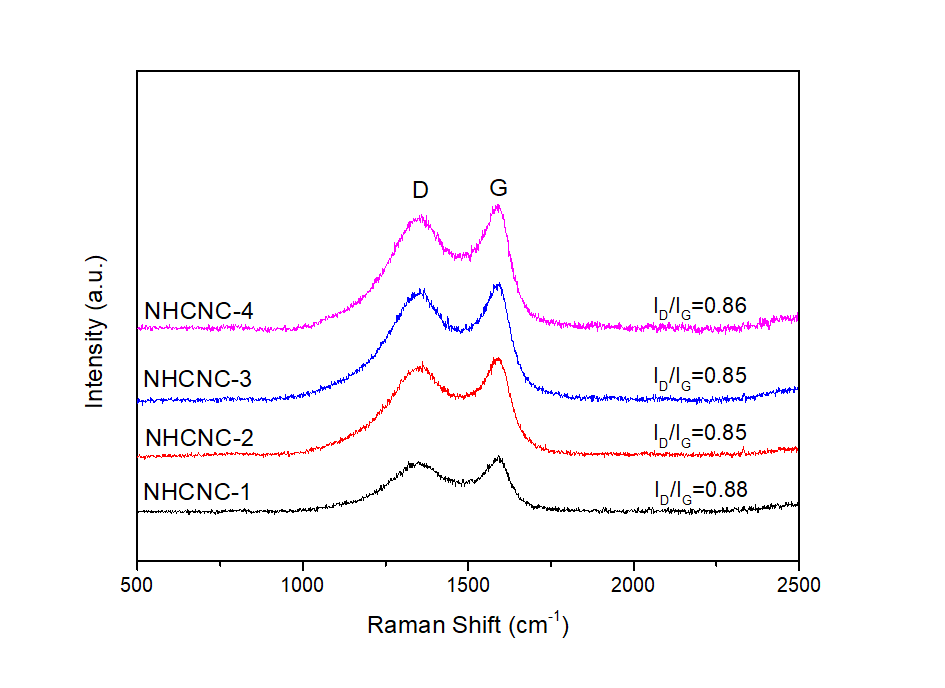


**Figure S2.** The Raman spectrum of NHCNCs, two intense peaks located at about 1332 cm^−1^ (D band) and 1586 cm^−1^ (G band) are attributed to *sp*^2^-based carbons with a graphitic character but possessing a high density of amorphous structure. Due to the increase in shell thickness, it is more difficult to form a uniform graphitized structure during the same calcination process, resulting in a higher *I_D_/I_G_* ratio (0.88) of NHCNC-1 than others. However, for NHCNC-4 which has the thinnest shell, the *I_D_/I_G_* ratio is 0.86, even higher than NHCNC-3 (0.85), implying more induced defects by N-doping.

**3. Electrochemistry performances of the supercapacitor devices with NHCNCs**


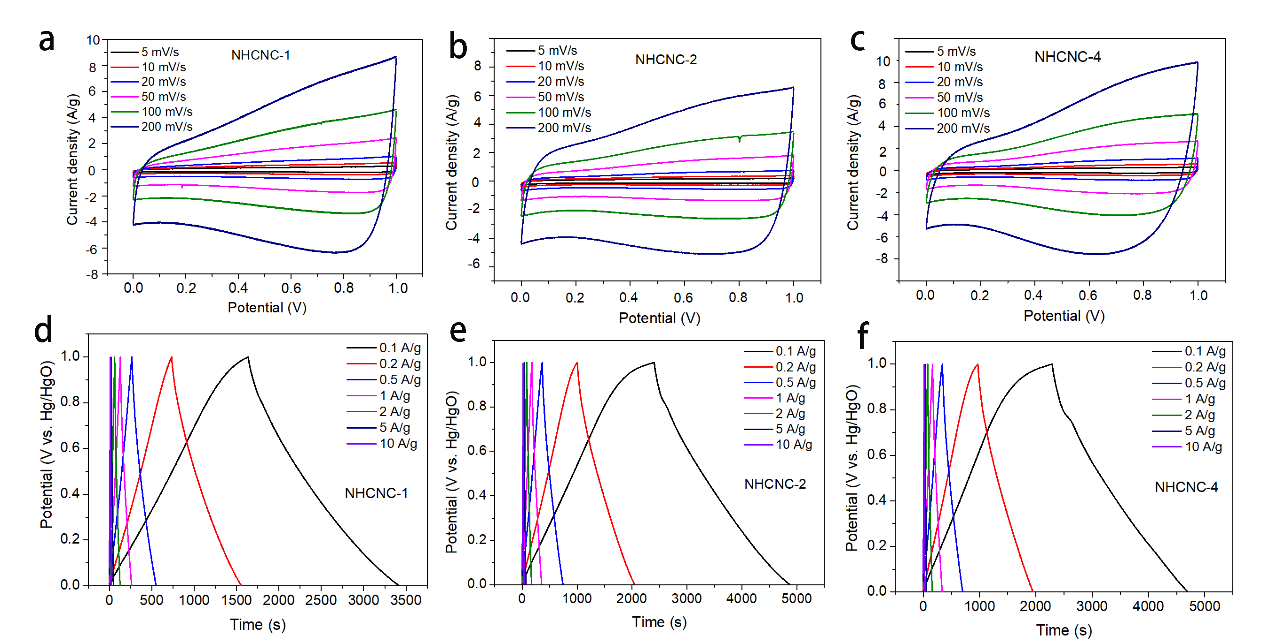


**Figure S3.** (a-c) CV curves at the various scan rates and (d-f) GCD curves at versus charge/discharge current density for NHCNC-1, NHCNC-2 and NHCNC-4.

**4. A photograph of 100 mg of NHCNC samples tapped in quartz tubes**


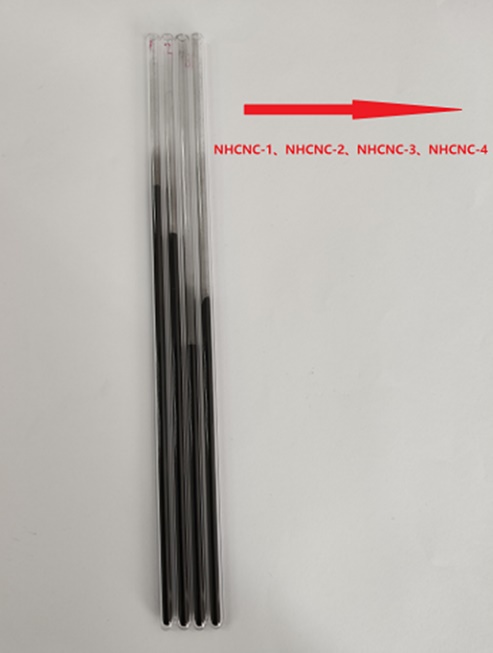


**Figure S4.** A photograph of 100 mg NHCNCs samples tapped in quartz tubes. The tap densities were evaluated by placing 100 mg of NHCNCs samples into a dry measuring cylinder, then taped hundreds of times. Clearly, the as-synthesized NHCNC-3 with semi-concave structure largely increase their packing density because of the much smaller empty cavities, which may result in higher volumetric capacitance or volume reduction of electrodes.

**5. The cytotoxicity of hollow carbon nanospheres on Hela cell line**

*Cytotoxicity evaluation:* The Hela cell line used as cytotoxicity evaluation model, was obtained from the American Type Culture Collection. The cells were maintained and cultured in Dulbecco’s modified Eagle’s medium. (DMEM, Hyclone), supplemented with 10% fetal bovine serum (FBS, Hyclone), 100 unit/mL penicillin (Gibco) and 100 μg/mL streptomycin (Gibco). Hela cells were cultured at 37 °C in a humidified atmosphere with 5% CO_2_ and were maintained as previously described with minor change.^1^ To measure cell viability, 5×10^3^ cells per well were seeded in a 96-well plate, cultured for 24 hours, and treated with different concentrations of the samples to be tested for 48 h. Cell viability was determined using an 3-(4,5-dimethylthiazol-2-yl)-2,5-diphenyltetrazolium bromide (MTT) assay kit (Sigma) as previously report.^1^ The proportion of viable cells was evaluated by normalizing the absorbance from the sample well against that from the control well and expressed as a percentage, with 100% assigned to the viability of Blank group cells.

As shown in **Fig. S5a** and S**5b**, both of NHCNC-1 and NHCNC-3 show no significant cytotoxicity on HeLa cells after 24 h of co-incubation. In general, the cell viability decreases slightly with an increase in concentration of nanomaterials, but the cell viabilities remain above 90% even though NHCHCs at dose as high as 512 µg/mL. Meanwhile, we have also examined the status of cell growth where they are treated with NHCHCs at 512 µg/mL. As shown in **Fig. S5c**, the NHCNCs-treated cells remained intact structure, density and shapes on the cell culture dish, and display similar growing status comparing blanks groups (un-treated cell groups). Without any cell debris and damage cells were observed in NHCHCs-treated groups.


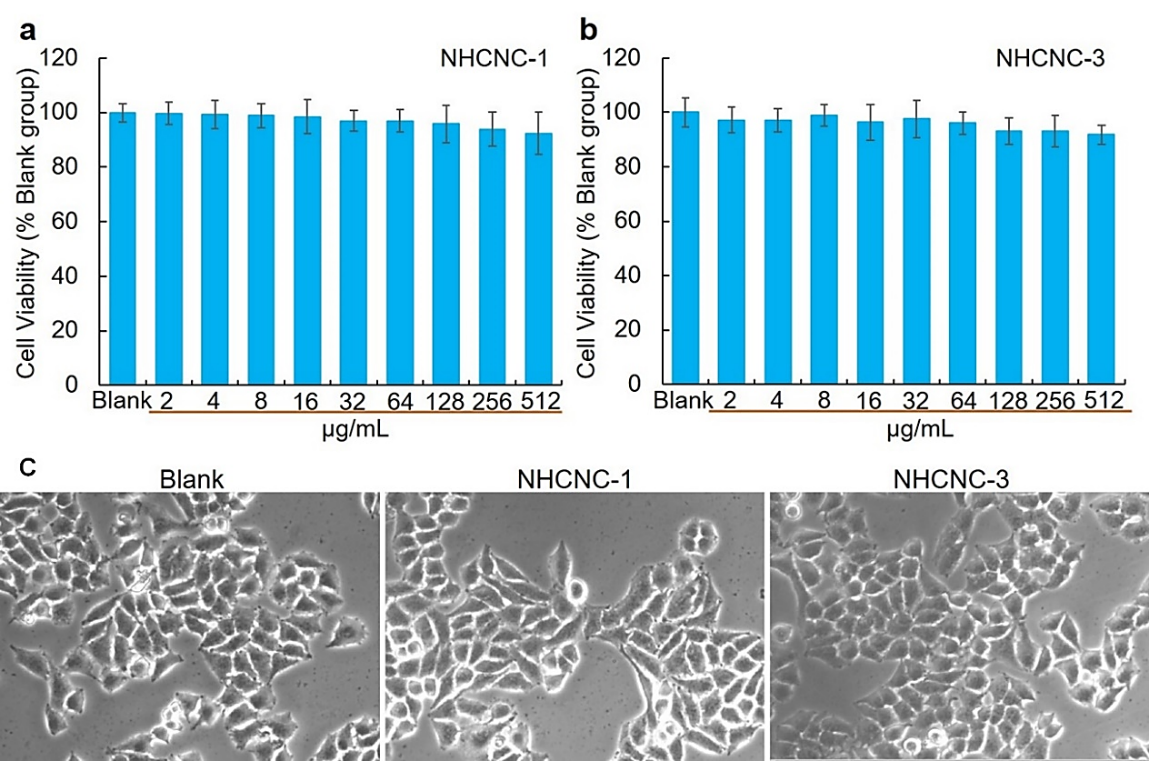


**Figure S5.** The cytotoxicity of hollow carbon nanospheres on Hela cell line. The cells were treated with different concentrations of (a) NHCNC-1 and (b) NHCNC-3 for 24 hours, and the intrinsic cytotoxicity was evaluated by the MTT assay. Blank group were untreated cells as negative control. The results are represented as means ± SD, n = 6. (c) Optical microscopy images of Hela cells taken after treated with NHCNCs for 24 h. The cell growth status and shape were acquired in different treated groups.

**6. Zeta potential of the prepared NHCNCs**

**Figure S6.** Zeta potentials for NHCNC-1, NHCNC-2, NHCNC-3 and NHCNC-4 (24.21 mV, 14.87 mV, 10.4 mV and 20.35 mV, respectively), clearly confirming that the NHCNCs are biocompatibility and not cytotoxic.

**7. Textural parameters, density and elemental compositions**

| **Samples** | **N_2_ adsorption-desorption** | | | **Shell thickness (nm)** | **Volumetric density**  **(gcm**^−3^**)** | **XPS analysis** | | |
| --- | --- | --- | --- | --- | --- | --- | --- | --- |
|  | **S_BET_ (m^2^g**^−1^**)** | **Pore diameter (nm)** | **V_total_**  **(cm^3^g**^−1^**)** |  |  | **C (at%)** | **N**  **(at%)** | **O (at%)** |
| **NHCNC-1** | 868.89 | 2.05 | 0.3938 | 15 | 0.96 | 90.51 | 2.84 | 6.65 |
| **NHCNC-2** | 1065.16 | 2.08 | 0.9224 | 10 | 1.02 | 89.58 | 3.45 | 6.97 |
| **NHCNC-3** | 1400.96 | 2.25 | 1.6079 | 10 | 1.41 | 89.46 | 3.73 | 6.81 |
| **NHCNC-4** | 1339.76 | 2.52 | 1.1677 | 7 | 1.26 | 86.77 | 4.51 | 8.72 |

**Table S1.** Textural parameters and elemental compositions of NHCNC-1, NHCNC-2, NHCNC-3 and NHCNC-4

**8. Comparison of the NHCNCs with previously reported carbon materials**

| **Materials** | ***C_g_***  **(F g^−1^)** | ***C_v_***  **(F cm^−3^)** | **Scan rate** | **Electrolytes** | **Ref** |
| --- | --- | --- | --- | --- | --- |
| Porous carbon | 267 | 113 | 0.5 A g^−1^ | 6M KOH | 2 |
| N-doped porous carbon | 298 | 161 | 0.20 A g^−1^ | 1M H_2_SO_4_ | 3 |
| B/N porous carbon | 247 | 101 | 0.50 A g^−1^ | 6M KOH | 4 |
| N/P co-doped carbon | 206 | 261 | 0.50 A g^−1^ | 1M H_2_SO_4_ | 5 |
| Carbon nanosheets | 233 | 177 | 0.1 A g^−1^ | 1M H_2_SO_4_ | 6 |
| Porous carbon shell | 251 | 182 | 1 A g^−1^ | 6M KOH | 7 |
| Carbon xerogel | 251 | 166 | 0.125 A g^−1^ | 1M H_2_SO_4_ | 8 |
| Mesoporous carbon | 171 | 107 | 5 mV·s^−1^ | 6M KOH | 9 |
| N-doped carbon fiber | 202 | 200 | 1 A g^−1^ | 6M KOH | 10 |
| CNFs | 280 | 88 | 0.50 A g^−1^ | 6M KOH | 11 |
| MWNTs | 159 | 132 | 50 mV s^−1^ | 1M H_2_SO_4_ | 12 |
| RGO | 285 | 218 | 1 A g^−1^ | 6M KOH | 13 |
| High-density graphene | 238 | 376 | 0.1 A g^−1^ | 6M KOH | 14 |
| N-modified FLG | 227 | 155 | 1 A g^−1^ | 6M KOH | 15 |
| RGO film | 180 | 226 | 5 mV s^−1^ | 6M KOH | 16 |
| HRGO-10 film | 251 | 216 | 1 A g^−1^ | 6M KOH | 17 |
| 3D porous carbon | 318 | 118 | 0.50 A g^−1^ | 6M KOH | 18 |
| Porous bulk | 383 | 165 | 0.2 A g^−1^ | 6 M KOH | 19 |
| Activated carbon xerogel | 251 | 166 | 0.125 A g^−1^ | 1M H_2_SO_4_ | 8 |
| carbon aerogels | 220 | 123 | 0.125 A g^−1^ | 1M H_2_SO_4_ | 20 |
| GO hydrogel | 133.6 | 176.5 | 1 A g^−1^ | 6M KOH | 21 |
| Holey GF | 208 | 148 | 1 A g^−1^ | 6M KOH | 22 |
| Graphene hydrogel | 203.9 | 293.6 | 0.5 A g^−1^ | 6M KOH | 23 |
| Graphene/CNT film | 175 | 160 | 50 mV s^−1^ | 0.5MH_2_SO_4_ | 24 |
| **Hollow Carbon Nanocapsule (NHCNC-3)** | **326** | **419** | 0.1 A g^−1^ | 6M KOH | **This study** |

**Table S2.** Comparison of the NHCNC with previously reported carbon materials.

# References

1. Yang, C. B. et al. Biodegradable nanocarriers for small interfering ribonucleic acid (siRNA) co-delivery strategy increase the chemosensitivity of pancreatic cancer cells to gemcitabine. *Nano Res.* **10**, 3049–3067 (2017).
2. Zheng, X. Y. et al. Oriented and interlinked porous carbon nanosheets with an extraordinary capacitive performance. *Chem. Mater.* **26**, 6896–6903 (2014).
3. Hao, L. et al. Terephthalonitrile-derived nitrogen-rich networks for high performance supercapacitors. *Energy Environ. Sci.* **5**, 9747–9751 (2012).
4. Guo, D. C. et al. Ionic liquid C_16_mimBF_4_ assisted synthesis of poly(benzoxazine-co-resol)-based hierarchically porous carbons with superior performance in supercapacitors. *Energy Environ. Sci.* **6**, 652-659 (2013).
5. Yan, X. D. et al. Simple and scalable synthesis of phosphorus and nitrogen enriched porous carbons with high volumetric capacitance. *Electrochim. Acta* **136**, 466–472 (2014).
6. Ling, Z. et al. Sustainable synthesis and assembly of biomass-derived B/N co-doped carbon nanosheets with ultrahigh aspect ratio for high-performance supercapacitors. *Adv. Funct. Mater.* **26**, 111–119 (2016).
7. Yang, W. et al. Template-free synthesis of ultrathin porous carbon shell with excellent conductivity for high-rate supercapacitors. *Carbon* **111**, 419–427 (2017).
8. Zapata-Benabithe, Z., Carrasco-Marin, F., de Vicente, J. & Moreno-Castilla, C. Carbon xerogel microspheres and monoliths from resorcinol-formaldehyde mixtures with varying dilution ratios: preparation, surface characteristics, and electrochemical double-layer capacitances. *Langmuir* **29**, 6166–6173 (2013).
9. Yu, X. L., Wang, J. G., Huang, Z. H., Shen, W. C. & Kang, F. Y. Ordered mesoporous carbon nanospheres as electrode materials for high-performance supercapacitors. *Electrochem. Commun.* **36**, 66–70 (2013).
10. Yan, J. et al. Template-assisted low temperature synthesis of functionalized graphene for ultrahigh volumetric performance supercapacitors. *ACS Nano* **8**, 4720–4729 (2014).
11. Li, W. et al. A Self-template strategy for the synthesis of mesoporous carbon nanofibers as advanced supercapacitor electrodes. *Adv. Energy Mater.* **1**, 382–386 (2011).
12. Lee, S. W., Kim, B. S., Chen, S., Shao-Horn, Y. & Hammond, P.T. Layer-by-layer assembly of all carbon nanotube ultrathin films for electrochemical applications. *J. Am. Chem. Soc.* **131**, 671–679 (2009).
13. She, Z., Ghosh, D. & Pope, M. A. Decorating graphene oxide with ionic liquid nanodroplets: an approach leading to energy-dense, high-voltage supercapacitors. *ACS Nano* **11**, 10077–10087 (2017).
14. Tao, Y. et al. Towards ultrahigh volumetric capacitance: graphene derived highly dense but porous carbons for supercapacitors. *Sci. Rep.* **3**, 2975 (2013).
15. Xiao, N. et al. A simple process to prepare nitrogen-modified few-layer graphene for a supercapacitor electrode. *Carbon* **57**, 184–190 (2013).
16. Jiang, L. L., Sheng, L. Z., Long, C. L. & Fan, Z. J. Densely packed graphene nanomesh-carbon nanotube hybrid film for ultra-high volumetric performance supercapacitors. *Nano Energy* **11**, 471–480 (2015).
17. Bai, Y. L. et al. Formation process of holey graphene and its assembled binder-free film electrode with high volumetric capacitance. *Electrochimica. Acta* **187**, 543–551 (2016).
18. Qie, L. et al. Synthesis of functionalized 3D hierarchical porous carbon for high-performance supercapacitors. *Energy Environ. Sci.* **6**, 2497–2504 (2013).
19. Zhao, J. et al. Hydrophilic Hierarchical nitrogen-doped carbon nanocages for ultrahigh supercapacitive performance. *Adv. Mater.* **27**, 3541–3545 (2015).
20. Zapata-Benabithe, Z., Carrasco-Marin, F. & Moreno-Castilla, C. Preparation, surface characteristics, and electrochemical double-layer capacitance of KOH-activated carbon aerogels and their O- and N-doped derivatives. *J. Power. Sources* **219**, 80–88 (2012).
21. Pham, V. H. & Dickerson, J. H. Reduced graphene oxide hydrogels deposited in nickel foam for supercapacitor applications: toward high volumetric capacitance. *J. Phys. Chem. C* **120**, 5353–5360 (2016).
22. Xu, Y. X. et al. Holey graphene frameworks for highly efficient capacitive energy storage. *Nat. Commun.* **5**, 4554; 10.1038/ncomms5554 (2014).
23. Tan, Y. Y. et al. Facile synthesis of functionalized graphene hydrogel for high performance supercapacitor with high volumetric capacitance and ultralong cycling stability. *Appl. Surf. Sci.* **455**, 683–695 (2018).
24. Byon, H. R., Lee, S. W., Chen, S., Hammond, P. T. & Shao-Horn, Y. Thin films of carbon nanotubes and chemically reduced graphenes for electrochemical micro-capacitors. *Carbon* **49**, 457–467 (2011).
